# *Enterococcus faecalis* NADH Peroxidase-Defective Mutants Stain Falsely in Colony Zymogram Assay for Extracellular Electron Transfer to Ferric Ions

**DOI:** 10.3390/microorganisms11010106

**Published:** 2022-12-31

**Authors:** Lars Hederstedt

**Affiliations:** The Microbiology Group, Department of Biology, Lund University, Sölvegatan 21, SE 223 62 Lund, Sweden; lars.hederstedt@biol.lu.se

**Keywords:** *Enterococcus faecalis*, EetB, Npr, heme, cytochrome *bd*, ferric reductase, Ferrozine

## Abstract

*Enterococcus faecalis* cells can reduce ferric ions and other electron acceptors by extracellular electron transfer (EET). To find mutants with enhanced or defective EET, strain OG1RF with random transposon insertions in the chromosome was screened for ferric reductase activity by colony zymogram staining using the chromogenic ferrous-chelating compound Ferrozine. The screen revealed *npr*, *eetB*, and *ndh3* mutants. The aberrant ferric reductase phenotype of Npr (NADH peroxidase)-defective mutants was found to be a property of colonies and not apparent with washed cells grown in liquid culture. EetB- and Ndh3-defective mutants, in contrast, consistently showed low ferric reductase activity. It is concluded that colony zymogram staining for ferric reductase activity using Ferrozine can be misleading, especially through false negative results. It is suggested that hydrogen peroxide produced in the colony quenches the zymogram staining. In addition, it is demonstrated that the negative effect of heme on EET to ferric ion in *E. faecalis* is relieved by cytochrome *bd* deficiency. The findings can help to identify bacteria with EET ability and contribute to our understanding of EET in Gram-positive bacteria and the physiology of *E. faecalis*.

## 1. Introduction

*Enterococcus faecalis* is a well-characterized, Gram-positive, opportunistic human pathogen [1]. This bacterium basically has a fermentative metabolism and cannot synthesize protoheme IX [2]. Similar to several other lactic acid bacteria, when provided with heme from the environment, it can respire with molecular oxygen by the oxidation of NADH in a simple electron transfer chain composed of a type II NADH:menaquinone oxidoreductase, demethylmenaquinone (DMK), and the menaquinol oxidase cytochrome *bd* [3,4]. *E. faecalis* cells can also transfer electrons from the oxidation of NADH in the cytoplasm to extracellular redox compounds, for example, ferric ions [5], and directly to an anode electrode [6,7,8]. This process is called extracellular electron transfer (EET). Compared to Gram-negative bacteria, such as *Geobacter* and *Shewanella* species, little is known about the molecular mechanisms behind the use of iron as electron acceptor in Gram-positive bacteria [9]. Understanding EET in microorganisms is of broad importance, for example, in the fields of ecology, medicine, and technology [10,11]. Regarding *E. faecalis*, EET might contribute to pathogenicity in the gut [1] and be of use in bioelectrochemical applications [12,13].

EET by aerobic *E. faecalis* cells is attenuated if respiration is active as the result of heme in the growth medium [7]. This is presumably because cytoplasmic NADH is more efficiently oxidized via the respiratory chain compared to via EET [7,14]. With the aim of finding mutants deficient in respiration, or for other reasons having enhanced EET, I have screened a library of *E. faecalis* strain OG1RF transposon-insertion mutants for high ferric reductase activity after growth in the presence of heme. The ferric reductase activity of colonies was determined using Ferrozine (3-(2-pyridyl)-5,6-bis(4-phenylsulfonic acid)-1,2,4-triazine monosodium hydrate) [15] as a reporter. This compound changes color from slightly yellow to intense magenta when complexed with Fe^2+^, the product of ferric reductase activity. The ferrous complex with Ferrozine is water-soluble and stable within the pH range 4 to 9 [15].

The screen identified mutants defective in NADH peroxidase, a cytoplasmic flavoenzyme that uses hydrogen peroxide to oxidize NADH, forming NAD^+^ and water as the products. The zymogram-staining activity of colonies of these mutants was in comparison to the parental strain high on plates supplemented with heme and low in the absence of heme. In addition, the screen led to the identification of an EetB and a Ndh3-deficient mutant with low zymogram-staining activity irrespective of heme in the plates.

## 2. Materials and Methods

### 2.1. Bacterial Strains and Growth Media

*E. faecalis* LFR20, LFR21, LFR24, and LFR30 are transposon-insertion mutants of OG1RF identified in this work. The origin and properties of EMB17 (*katA*::Tn) and EMB44 (Δ*cydABCD*), and of WY84 (Δ*menB*), are described in references [16,17], respectively. For reference to the wild-type strains OG1RF and JH2-2, see [18,19]. The construction of the library of clones with EfaMarTn inserted into the chromosome of strain OG1RF has been described before [16] and individual clones were stored as stock frozen at −80 °C in 25% glycerol in 96-microwell trays. For the screening of transposon-insertion mutants, clones were replica plated from the microwells to 8.5 cm diameter agar plates using a homemade square-sized grid with 48 nails. Thus, two agar plates were used to screen all of the clones of each microwell tray.

Strains were grown on tryptose soy broth (without dextrose; Difco Co., Beyrouth, Lebanon) (TSB) supplemented with 1% (*w*/*v*) glucose (TSBG) or 0.3% (*w*/*v*) glycerol. For the plates, 1.8% (*w*/*v*) agar was added. TSB contains less than 0.05 µM heme [20]. Depending on the experiment, the medium was supplemented with hemin (added from a 10 mM stock solution in dimethyl sulfoxide) and 0.2 mM ammonium iron(III) sulfate dodecahydrate, as indicated in the Results section. The H_2_O_2_ concentrations in the spent growth media were determined using the FOX1 reagent [21] and Peroxide 100 test sticks (Quantofix^®^; Fluka Chemie GmbH, Buchs, Switzerland).

### 2.2. Colony Zymogram Staining for Ferric Reductase Activity

Colony Ferrozine zymogram staining was conducted as described before [14]. Colonies were grown overnight at 37 °C on 5 or 8.5 cm diameter plastic Petri dishes containing 10 mL and 20 mL agar growth medium, respectively. For the zymogram staining, 5 and 10 mL, respectively, of a soft agar overlay containing 2 mM Ferrozine were applied on the plates. The magenta color development after the application of the overlay was registered during incubation at room temperature for up to two hours. The parental strain OG1RF was included on the plates as a standard for wild-type staining intensity.

### 2.3. Genetic Characterization of Mutants

The transposon-insertion site in chromosomal DNA was identified using inverse PCR and DNA sequence analysis [16]. The insertion sites were confirmed by PCR. The following oligonucleotides were used as primers in the sequence analysis and PCR in addition to those used before [14]: eetAB_up, 5‘-GCTAGATACGGCGGTAAG; eetAB_dw, 5‘-CGTAGGATAGGTATTCCACAT; npr_up, 5‘-GGCGAAATACAGAGG; npr_dw, 5‘-CTCGTGTCCAACAAG.

### 2.4. Determination of Ferric Reductase Activity of Washed Cells

The bacteria were grown in 10 mL TSBG in 100 mL glass Erlenmeyer flasks at 200 rpm and 37 °C. When in the mid-exponential growth phase, the cells were harvested by centrifugation at room temperature. After a wash in 2 mL TSB, the cells were diluted in TSB to an optical density at 600 nm of 1.2 and stored in an ice bath until use the same day. The ferric reductase activity was determined at 37 °C in the presence of 1 mM ammonium iron(III) sulfate, 2 mM Ferrozine, 0.4% (*w*/*v*) glucose, following the increase in absorption at 562 nm using 1 mL cuvettes and a double beam spectrophotometer as described before [14]. The procedure is based on the method of Deneer and Boychuk [22]. The ferric reductase activity of the cells grown in liquid TSGB did not differ from that of the cells grown in this medium supplemented with 0.2 mM ammonium iron(III) sulfate.

## 3. Results

### 3.1. Screen for Mutants

**Figure 1:**
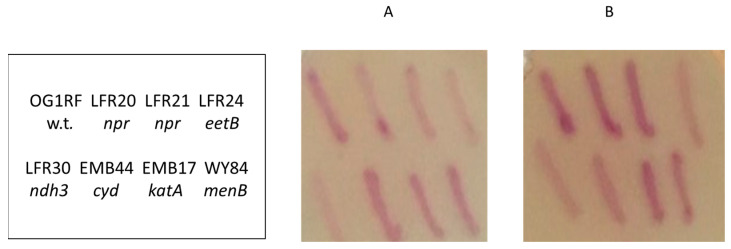
Colony ferric reductase activity of *E. faecalis* strains determined by Ferrozine zymogram staining. Panel (**A**), TSBG plate supplemented with 0.2 mM ammonium iron(III) sulfate. Panel (**B**), same medium as in Panel (**A**) and with 5 µM hemin added. The identity of the strains grown as streaks on the plates and their respective gene defect are indicated in the left-hand panel.

A library of clones with single EfaMarTn insertions in the chromosome of strain OG1RF were used to find *E. faecalis* mutants with increased or decreased EET activity. A total of 1286 different clones, stored as frozen cultures in microwell trays, were replica-plated onto TSBG agar plates supplemented with 0.2 mM ammonium iron(III) sulfate and 5 µM hemin, as described in the Materials and Methods section. At the used concentration, hemin does not affect growth and saturates the cells for heme, as shown before [20,23] and confirmed in this study using TSBG plates supplemented with ferric ions (data not shown). After growth overnight at 37 °C, the colonies on the plates were stained for ferric reductase activity using a soft agar overlay containing 2 mM Ferrozine. The clones found to stain differently compared to the parental strain were then taken from the original frozen stock cultures and patched onto the same kind of plate used for the screen to confirm the phenotype. They were also patched onto plates not containing hemin to determine the effect of heme on the staining activity. After growth overnight, the colonies on the plates were zymogram-stained for ferric reductase activity (Figure 1). The screen resulted in the isolation of four clones: LFR20, LFR21, LFR24, and LFR30 (Figure 1).

Clones LFR20 and LFR21 presented a somewhat stronger staining activity than the parental strain after growth in the presence of heme (Figure 1; panel B), but weak activity in the absence of heme (Figure 1; panel A). The two other clones, LFR24 and LFR30, stained weaker than the parental strain independent of hemin in the growth medium. The used library of transposon-insertion mutants has been used before to find mutants defective in EET when grown without heme [14], but the four clones were not found in that work. The phenotype of LFR20 and LFR21 colonies, with strong staining in the presence of heme and weak staining in the absence of heme, is novel (Table 1).

### 3.2. npr-Defective Mutants

**Figure 2:**
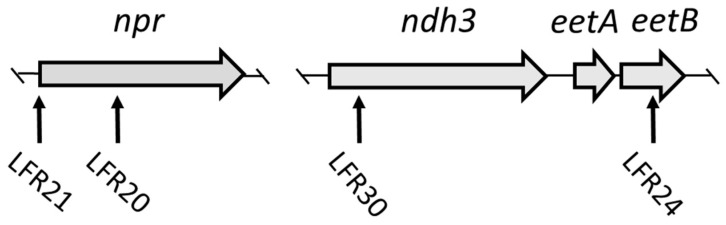
Map of the genes at two loci in the chromosome of *E. faecalis*. The transposon insertion position in the four mutants identified in this work are indicated with vertical arrows. For additional information, see Table 2.

Clones LFR20 and LFR21, with the novel staining phenotype, were found to have the transposon inserted into the *npr* gene (Figure 2 and Table 2). In LFR20 the *npr* open reading frame is disrupted. In LFR21, the transcription of the *npr* gene is blocked due to the insertion of the transposon 22 base pairs upstream of the ATG translation initiation codon. The *npr* gene encodes NADH peroxidase (Npr) which has a major role in the detoxification of H_2_O_2_ in the cytoplasm of *E. faecalis* [24].

The analysis of five other OG1RF *npr*-defective strains (EMB7, EMB8, EMB15, EMB20, and EMB37), and also an *npr* deletion mutant of the laboratory strain JH2-2 [24], demonstrated that the novel staining phenotype is general for *E. faecalis* mutants defective in NADH peroxidase (Table 1). Interestingly, these five tested OG1RF mutants were found in a study comprising the isolation of mutants defective in catalase (KatA) activity [16]. In that study, it was demonstrated that *npr*-defective mutants contain inactive catalase protein. The reason for this enzyme defect remains enigmatic, but presumably the catalase is inactivated by the high cytoplasmic concentrations of H_2_O_2_ that result from the lack of NADH peroxidase activity [25] (Baureder and Hederstedt, unpublished data). Important for the present work, the colonies of *E. faecalis* strains without catalase protein (EMB1 and EMB17) showed the same pattern of colony ferric reductase zymogram staining as the parental strain on plates both with and without hemin (Figure 1) (Table 1). This observation ruled out that catalase deficiency is the cause of the novel Ferrozine colony staining phenotype of *npr*-defective mutants.

### 3.3. LFR24 and LFR30 Are Defective in EET to Ferric Ions

**Figure 3:**
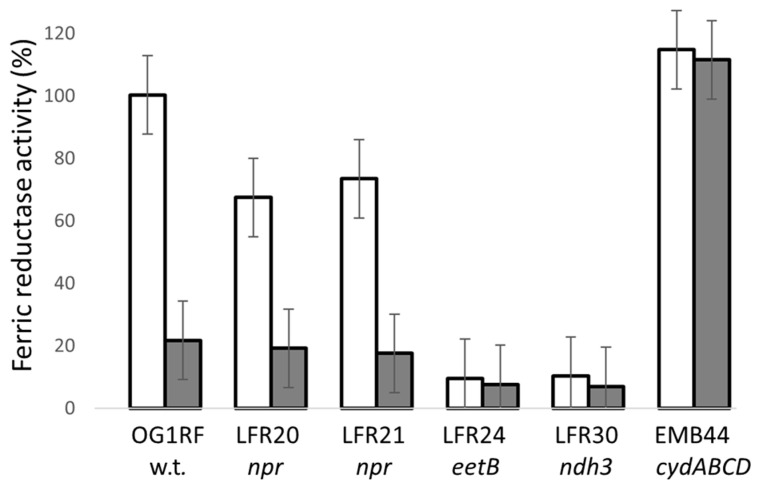
Ferric reductase activity of washed cells. The bacteria were grown in TSBG (open bar) or TSBG supplemented with 8 µM hemin (filled bar). The activity for each strain and growth condition was determined in at least two independent experiments and with two measurements per sample. The mean activity and standard deviation are presented.

Clone LFR24 was found to have the *eetB* gene disrupted by the transposon, whereas in LFR30, the *ndh3* gene is inactivated (Figure 2 and Table 2). The weak staining phenotype of LFR24 and LFR30 colonies on plates not supplemented with hemin is as expected, because Ndh3 is known and EetB predicted to be important for ferric reductase activity under these conditions [14]. Washed cells of LFR24 and LFR30 grown in liquid TSBG showed about 10% ferric reductase activity compared to the parental strain (Figure 3). This low activity is similar to that of Ndh3- and EetA-defective mutants [14] and confirmed the colony Ferrozine-staining phenotype of LFR24 and LFR30 on TSBG plates. Ndh3 is a NADH:menaquinone oxidoreductase, providing reducing equivalents for EET, and EetB together with EetA are membrane proteins important for ferric ion-dependent EET in *E. faecalis* and other bacteria [14,26,27].

### 3.4. NADH Peroxidase Mutants Have Ferric Reductase Activity

The washed cells of the *npr*-defective mutants LFR20 and LFR21 grown in TSBG showed about 70% ferric reductase activity compared to the parental strain (Figure 3). It is a much higher activity than that of the ferric reductase-defective mutants LFR24 and LFR30. The supplementation of the growth medium with hemin attenuated the ferric reductase activity of LFR20 and LFR21, just as for the parental strain OG1RF (Figure 3). The results showed that *npr*-defective *E. faecalis* strains have ferric reductase activity, despite the weak Ferrozine zymogram-staining activity observed for colonies after growth in the absence of heme and the strong staining after growth in the presence of heme (Figure 1) (Table 1). Thus, the colony zymogram-staining and the enzyme activity of the washed cells did not agree for the *npr*-defective mutants.

### 3.5. Ferrozine Zymogram-Staining Activity of Colonies Depends on Multiple Factors

To explore the inconsistencies observed between the results with the colony zymogram stain and ferric reductase activity of washed cells, I analyzed the properties of strains WY84 and EMB44. WY84 (Δ*menB*) is blocked in the synthesis of DMK and therefore defective in ferric reductase activity independent of hemin in the medium [7,14]. Strain EMB44 (Δ*cydABCD*) lacks cytochrome *bd* and washed cells showed high ferric reductase activity also after growth with hemin (Figure 3). On the agar plates, the colonies of strain WY84 stained weaker than the parental strain, but stronger than the ferric reductase-defective mutants LFR24 and LFR30, both with and without hemin in the medium (Figure 1) (Table 1). The colonies of strain EMB44 stained as the parental strain in the absence of hemin, as expected, since cytochrome *bd* is not assembled in the parental strain under these conditions [3,7]. However, in the presence of hemin, the colonies of this mutant unexpectedly stained weaker than the parental strain (Figure 1; panel B). The findings with strains WY84 and EMB44 further indicated that the zymogram-staining activity of colonies do not always correlate with the ferric reductase activity of washed cells.

Ferric and ferrous ions and EET are known to affect *E. faecalis* biofilm features and therefore presumably the properties of colonies on agar plates [8,26]. Poor staining of colonies was obtained if ammonium iron(III) sulfate was not added to the TSBG growth medium of the agar plates or if only hemin was added (Figure S1). This confirmed that the zymogram-staining activity of the colonies is ferric ion-dependent. Heating the plates at 80 °C for 15 min before colony ferric reductase zymogram staining essentially abolished the color development (Figure S2). This result indicates that the observed staining of colonies mainly depends on ongoing enzyme activity and not, for example, on Fe^2+^ ions that have accumulated on the cells or in the vicinity of the cells in the colony during the overnight growth period on the plate.

The more intense staining activity of the colonies grown in the presence of hemin compared to without hemin (Figure 1) (Table 1) suggested that heme might mediate electron transfer from the cells to Fe^3+^. To analyze this possibility, the ferric reductase activity of washed OG1RF cells grown in TSBG were analyzed with 5 µM hemin added to the cuvette just before the assay was initiated. The activity decreased to about 20% compared to the control (which received the corresponding volume of dimethyl sulfoxide; the solvent for the hemin stock solution). However, the ferric reductase activity of washed cells grown in the presence of hemin was unaffected, as illustrated by the high ferric reductase of strain EMB44 grown in the presence of 8 µM hemin (Figure 3). The supplementation of the overlay staining solution with 10 µM hemin did not affect the staining intensity of colonies of the various strains grown on TSBG plates supplemented with Fe^3+^. Based on these findings, it is concluded that heme in the growth medium does not directly mediate the electron transfer from cells to Fe^3+^.

### 3.6. Hydrogen Peroxide Quenches in Ferric Reductase Assays

Large amounts (≤0.6 mM) of H_2_O_2_ can be found in the liquid culture supernatant after the growth of *E. faecalis* strains and related lactic acid bacteria, for example, *Lactobacillus casei*, if the cells are grown with glycerol and especially if NADH peroxidase is deficient [24,25,28,29]. Under aerobic conditions, glycerol can be metabolized in *E. faecalis* via two pathways: dependent on an NAD-dependent glycerol dehydrogenase and a glycerol kinase, respectively, that catalyzes the first step in the pathway. In the latter case, the oxygen-dependent enzyme glycerol-3-phosphate oxidase catalyzes the second step, resulting in the generation of one molecule of H_2_O_2_ per turnover of the enzyme. Strain JH2-2 has both pathways, whereas OG1RF seems to have only the pathway with glycerol-3-phosphate oxidase [28]. The cytoplasmic membrane is permeable to H_2_O_2_ and peroxide generated in the cytoplasm therefore leaks out of the cell if not decomposed by the activity of NADH peroxidase, catalase, and several other scavenging enzymes including cytochrome *bd* [30]. Superoxide in *E. faecalis* is also produced extracellularly and this process is attenuated when heme is available and dependent on the DMK in the membrane [31]. Superoxide can be dismutated into H_2_O_2_, and in the presence of metal ions, very reactive short-lived hydroxyl radicals are produced.

H_2_O_2_ is known to react with Fe^2+^ and reduced heme in Fenton and Fenton-like reactions [32]. The oxidation of Fe^2+^ would negatively interfere in the ferric reductase activity assay by removing enzyme products. To analyze this, H_2_O_2_ was added to the colony overlay zymogram staining solution. At 10 mM, a sublethal concentration for *E. faecalis* [33], the staining of colonies of the different strains was quenched (Figure S3). This effect of H_2_O_2_ was irrespective of whether the plates contained heme or not. H_2_O_2_ is not present in preparations of washed cells. The inclusion of 10 mM H_2_O_2_ in the ferric reductase activity assay with washed OG1RF cells blocked the color development: the activity was reduced 20-fold compared to without H_2_O_2_, and at 2 mM, it was reduced to about 50%. The ferrous Ferrozine complex was found stable after exposure to 15 mM H_2_O_2_, as determined by the absorption at 562 nm, indicating that the effect of peroxide occurs prior to complex formation. Thus, the quenching of zymogram staining by H_2_O_2_ produced by the cells might significantly contribute to the apparent poor ferric reductase activity of colonies.

The overnight culture supernatants of strain LFR20 grown in liquid TSB supplemented with 0.3% (*w*/*v*) glycerol were found to contain 25 to 50 µM H_2_O_2._ The corresponding culture supernatant of the parental strain and of LFR20 grown with glucose instead of glycerol contained < 10 µM H_2_O_2_ (below the detection limit). Consistent with the effect of H_2_O_2_ on the stain intensity, the colonies grown on plates containing glycerol instead of glucose showed low zymogram-staining activity (Figure S4). This was as expected if more H_2_O_2_ accumulates in the colonies during growth on glycerol.

## 4. Discussion

In this work, *npr*-, *eetB*-, and *ndh3*-defective *E. faecalis* mutants were isolated as the result of the screening of colonies on agar plates for Fe^3+^-dependent Ferrozine zymogram-staining activity. The phenotypic analysis of the isolates showed that the *eetB*- and *ndh3*-defective mutants are deficient in ferric reductase activity and the colonies stained weaker than the parental strain independent of the presence of heme in the growth medium. The *npr* mutants, defective in NADH peroxidase, showed almost wild-type ferric reductase activity and, despite this, stained weakly when grown on plates without heme. Thus, the results of the Ferrozine staining of colonies should be interpretated with caution, as illustrated by the false weak staining of *E. faecalis npr*-defective mutants after growth on TSBG agar plates. The Ferrozine zymogram colony staining procedure has been used to demonstrate the ferric reductase activity of bacteria and screen for mutants ([14,22,27], this work) and to find electrogenic bacterial species in the human skin and gut microbiota [34]. It is therefore important to acknowledge the limitations and potentials of this zymogram method.

**Figure 4:**
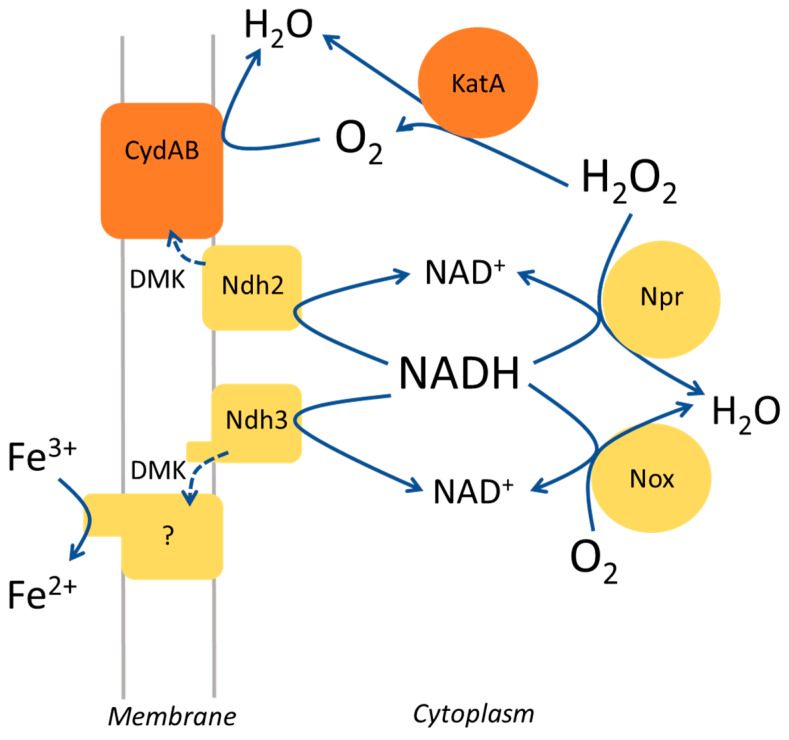
Schematic drawing presenting the roles of enzymes in *E. faecalis* relevant in this study. CydAB, cytochrome *bd*; DMK, dimethylmenaquinone; KatA, catalase; Npr, NADH peroxidase; Ndh, NADH dehydrogenase; Nox, NADH oxidase. Dotted lines indicate electron transfer. Cytochrome *bd* and catalase are only assembled and functional when heme is provided in the growth medium [4]. The function of the membrane-associated NADH dehydrogenase Ndh3 is devoted to EET, whereas that of Ndh2 seems to be in respiration with molecular oxygen [14]. Proteins that function in the electron transfer from reduced DMK to extracellular ferric ions are not known at present and this is indicated by a question mark.

The results presented in this study show that the ferric reductase activity of cells in the colony Ferrozine zymogram staining can be overshadowed by other activities linked to metabolism. One interfering component seems to be extracellular H_2_O_2_ that reacts with ferrous ion, the direct product of the ferric reductase activity. Fenton-like reactions with H_2_O_2_ can result in short-lived oxidative radicals that react with organic substances, causing, for example, damage to lipids and proteins in cells. The chemistry is complex and depends on the conditions, including the pH and how the iron ion is coordinated [32]. In *E. faecalis*, H_2_O_2_ is mainly degraded by NADH peroxidase and also when heme is available by catalase, but other peroxidases also contribute [24,25,35]. Consequently, in *E. faecalis*, several enzyme activities combined determine the amount of H_2_O_2_ that accumulates in the cytoplasm and on the outside of the cell (Figure 4). The NADH/NAD^+^ concentration ratio in the cytoplasm has a modulatory effect on the relative activities of NADH peroxidase, respiration with molecular oxygen, and EET dependent on Ndh3, because of the different affinity for NADH of the enzymes. The activities are also determined by the availability of heme, H_2_O_2_, and extracellular electron acceptors for EET. Notably, molecular oxygen is a product of catalase activity and is consumed by several enzymes in the cell, for example, the membrane-associated glycerol-3-phosphate oxidase and cytochrome *bd*, and the cytoplasmic NADH oxidase (Figure 4). Cytochrome *bd* has a very high affinity for oxygen and can operate under microaerophilic conditions [36]. The apparent higher colony Ferrozine-staining activity of the parental strain and *npr*-defective mutants when grown with hemin (Figure 1) might reflect a high NADH/NAD^+^ ratio in the cells combined with a low H_2_O_2_ concentration in the colony.

In the electron transfer from the cytoplasm to extracellular ferric ion, the oxidation of NADH by the flavoprotein Ndh3 is coupled to the reduction of DMK (or a modified variant of this naphtoquinone) in the membrane (Figure 4). At present, it is not known how electrons are transferred from the reduced menaquinone to ferric ion in *E. faecalis*. Originally, it was proposed that EetA, EetB, and PplA function in this transfer [27]. However, PplA is not required for EET to Fe^3+^ in *E. faecalis* [14] and recent experimental findings in *Listeria monocytogenes* show that EetB is a FAD-binding protein [37]. EetB is now suggested to be the substrate-binding component of an energy-coupled factor transporter that delivers FAD to FmnB, which is active on the outer side of the cytoplasmic membrane, catalyzing the covalent binding of FMN to a family of proteins [37,38]. The role of EetA is not known, but the protein seems tightly connected to the function of EetB [37]. In a previous study, Ndh3, DMK, and EetA were shown to be important for the ferric reductase activity of washed *E. faecalis* cells [14]. The present work demonstrates that EetB is also required, which is consistent with other studies [26,27,37]. The direct electron donor(s) to ferric ion in the cell envelope of *E. faecalis* and other Gram-positive electrogenic bacteria, such as *Listeria monocytogemes* and *Lactiplantibacillus plantarum*, is not known. It might be electron-conductive pili, one or more flavoproteins exposed on the outer side of the cytoplasmic membrane, and depend on small soluble redox compounds in the cell envelope [26,37,39]. The so-far-reported screens for ferric reductase-defective mutants of *L. monocytogenes* and *E. faecalis* based on colony Ferrozine zymogram staining have not identified any genes outside of the so called FLEET locus, comprising *pplA*, *eetAB*, and *ndh2/3*, and *dmkAB* genes for quinone synthesis [14,27] (this work). This suggests that possible additional genes required for EET to ferric ion are functionally redundant or essential for growth, or have, for other reasons, evaded detection in the used experimental approach.

The overall conclusion of the present study is that, in *E. faecalis*: (i) EetB is like Ndh3 required for ferric reductase activity, (ii) NADH peroxidase is not important for ferric reductase activity, (iii) the outcome of the Ferrozine zymogram-staining activity of colonies is influenced by multiple factors connected to metabolism and therefore can vary depending on the growth conditions, and (iv) when cytochrome *bd* is absent or defective, cells have the potential for high EET also when grown in the presence of heme. The last listed finding is of practical value, for example, in the optimization of microbial fuels cells relying on electrogenic lactic acid bacteria and a complex growth medium (that often contains heme) combined with a heterogeneous population of microbial species that might cross-feed heme or short-chain menaquinones or menaquinone synthesis precursors [40,41].

## Figures and Tables

**Table 1:** Summary of relative Ferrozine staining activity of colonies grown on TSBG plates supplemented with 0.2 mM ammonium iron(III) sulfate (Fe) and ±5 µM hemin. +, magenta stain intensity similar to the parental strain OG1RF grown without hemin; −, weak stain; +(−), intermediate stain; ++, strong stain; ++(+), stain stronger than for the parental strain grown with hemin.

Strain ^a^	Gene Defect	TSBG + Fe	TSBG + Fe + Hemin
OG1RF	(wild-type)	+	++
LFR20	*npr*	−	++(+)
LFR21	*npr*	−	++(+)
LFR24	*eetB*	−	−
LFR30	*ndh3*	−	−
EMB1	*katA*	+	++
EMB4	*cydC katA*	+	+(−)
EMB7	*npr*	−	++(+)
EMB8	*npr*	−	++(+)
EMB15	*npr*	−	++(+)
EMB17	*katA*	+	++
EMB20	*npr*	−	++(+)
EMB37	*npr*	−	++(+)
EMB44	*cydABCD*	+	+(−)
JH2-2	(wild-type)	+	++
JH2-2 Δnpr	*npr*	−	++(+)
*WY84*	*menB*	*+*(*−*)	*+*(*−*)

^a^ All mutant strains are derived from OG1RF, except JH2-2 Δnpr, which is a strain JH2-2 derivative.

**Table 2:** Genotype of *E. faecalis* mutants identified in this work.

Strain	Gene	EfaMarTn Insertion Position ^a^	Locus Tag
LFR20	*npr*	1.177.185	OG1RF_10983
LFR21	*npr*	1.176.588	OG1RF_10983
LFR24	*eetB*	3.137.739	OG1RF_12512
LFR30	*ndh3*	3.135.429	OG1RF_12510

^a^ GenBank accession no. CP002621.1.

## Data Availability

The strains isolated and characterized in this study are available from the author.

## Supplementary Materials

The following supporting information can be downloaded at: https://www.mdpi.com/article/10.3390/microorganisms11010106/s1, Figures S1–S4 showing Ferrozine zymogram staining of colonies for ferric reductase activity.
