# Supplementary material for: Enterococcus faecalis NADH Peroxidase-Defective Mutants Stain Falsely in Colony Zymogram Assay for Extracellular Electron Transfer to Ferric Ions
Source: Microorganisms. 2022 Dec 31;11(1):106. doi: 10.3390/microorganisms11010106 (PMC9864754; doi:10.3390/microorganisms11010106)
Supplement: Supplementary file 1 [file microorganisms-11-00106-s001.zip › microorganisms-2106931-supplementary.pdf]

Supplementary material

Enterococcus faecalis NADH peroxidase-defective mutants stain falsely in colony zymogram assay for extracellular electron transfer to ferric ions

Lars Hederstedt

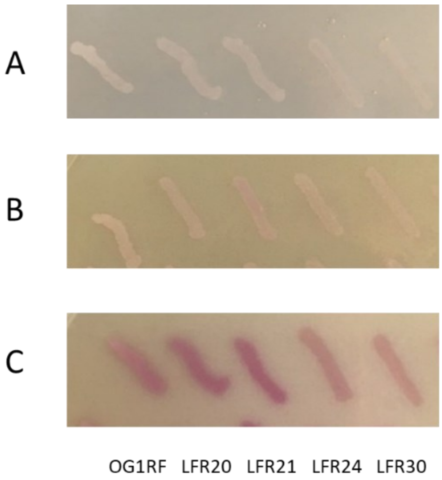

Figure S1. Ferrozine zymogram staining of colonies for ferric reductase activity. The five *E. faecalis* strains were grown on TSBG plates. **A**, without supplementation; **B**, with 5 µM hemin; **C**, with 0.2 mM ammonium iron(III) sulfate plus 5 µM hemin. After incubation over night the plates were stained using Ferrozine. The photos were taken after 30 min incubation at room temperature.

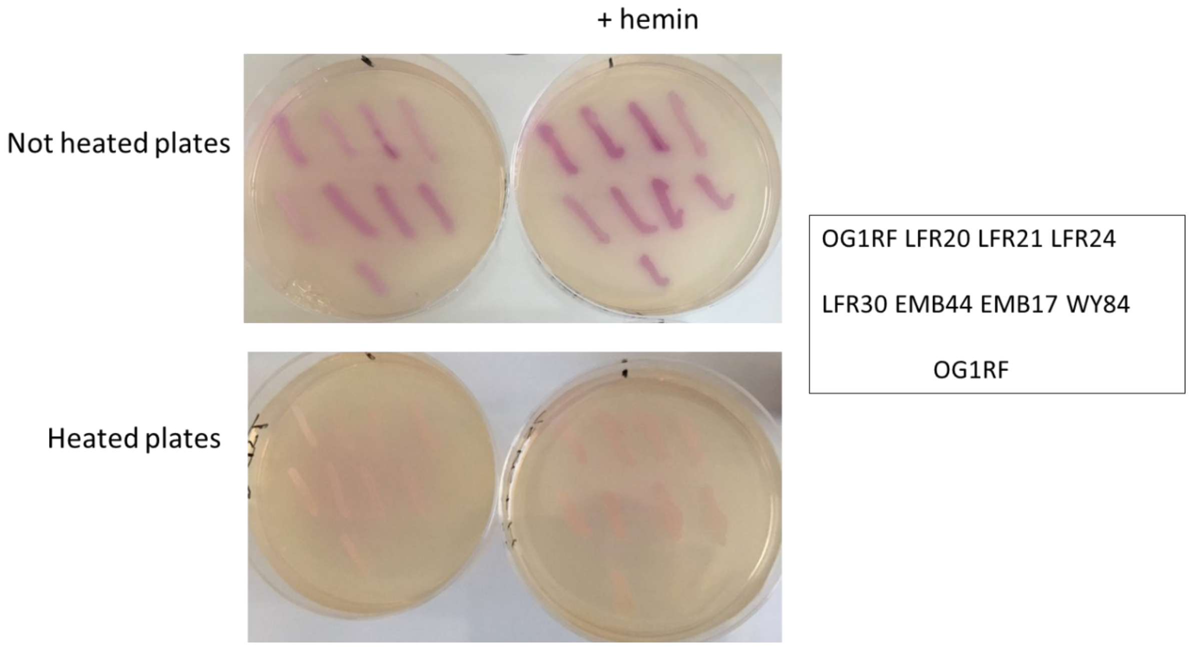

Figure S2. Ferrozine zymogram staining of colonies for ferric reductase activity. The bacteria were grown over night on two sets of TSBG plates containing 0.2 mM ammonium iron(III) sulfate and with and without 5 µM hemin. One set was heated at 80° C for 15 min on a water bath. All four plates

were then zymogram stained. Photos were taken after 30 min. The identity of the strains on the plates is shown in the right-hand side panel.

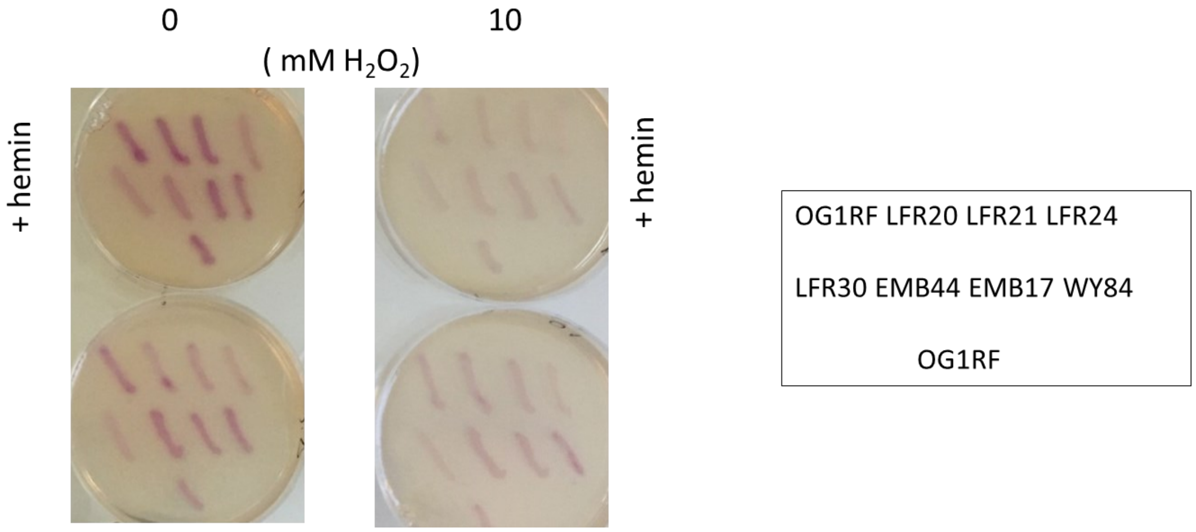

Figure S3. Ferrozine zymogram staining of colonies for ferric reductase activity. Bacteria were grown over night on two sets of TSBG plates containing 0.2 mM ammonium iron(III) sulfate and with and without 5 µM hemin as indicated. The four plates were then stained with and without 10 mM H_2O_2 added to the overlay stain solution. Photos were taken after 30 min.

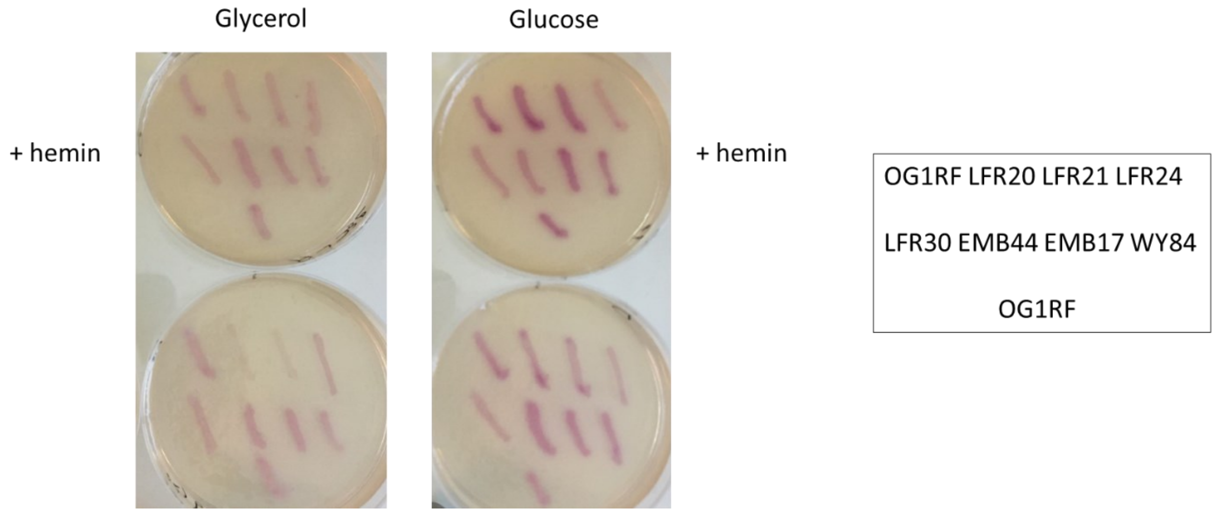

Figure S4. Ferrozine zymogram staining of colonies for ferric reductase activity. The indicated *E. faecalis* strains were grown over night on TSB plates containing 0.2 mM ammonium iron(III) sulfate and 0.3% glycerol or 0.5% glucose and with and without 5 µM hemin. Photos were taken after 30 min after adding the overlay soft agar stain.
